# Genome Sequencing and Analysis of *Trichoderma* (Hypocreaceae) Isolates Exhibiting Antagonistic Activity against the Papaya Dieback Pathogen, *Erwinia mallotivora*

**DOI:** 10.3390/jof8030246

**Published:** 2022-02-28

**Authors:** Amin-Asyraf Tamizi, Noriha Mat-Amin, Jack A. Weaver, Richard T. Olumakaiye, Muhamad Afiq Akbar, Sophie Jin, Hamidun Bunawan, Fabrizio Alberti

**Affiliations:** 1 Agri-Omics and Bioinformatics Programme, Biotechnology and Nanotechnology Research Centre, Malaysian Agricultural Research and Development Institute Headquarters (MARDI), Serdang 43400, Selangor, Malaysia; aminasyraf@mardi.gov.my (A.-A.T.); noriha@mardi.gov.my (N.M.-A.); 2 School of Life Sciences, University of Warwick, Gibbet Hill Road, Coventry CV4 7AL, UK; jack.weaver@warwick.ac.uk (J.A.W.); richard.olumakaiye@warwick.ac.uk (R.T.O.); sophie.jin@warwick.ac.uk (S.J.); 3 Institute of Systems Biology, Universiti Kebangsaan Malaysia (UKM), Bangi 43600, Selangor, Malaysia; muhdafiq.akbar@gmail.com

**Keywords:** biocontrol, fungus, *Trichoderma*, Gram-negative, *Erwinia*

## Abstract

*Erwinia mallotivora*, the causal agent of papaya dieback disease, is a devastating pathogen that has caused a tremendous decrease in Malaysian papaya export and affected papaya crops in neighbouring countries. A few studies on bacterial species capable of suppressing *E. mallotivora* have been reported, but the availability of antagonistic fungi remains unknown. In this study, mycelial suspensions from five rhizospheric *Trichoderma* isolates of Malaysian origin were found to exhibit notable antagonisms against *E. mallotivora* during co-cultivation. We further characterised three isolates, *Trichoderma koningiopsis* UKM-M-UW RA5, UKM-M-UW RA6, and UKM-M-UW RA3a, that showed significant growth inhibition zones on plate-based inhibition assays. A study of the genomes of the three strains through a combination of Oxford nanopore and Illumina sequencing technologies highlighted potential secondary metabolite pathways that might underpin their antimicrobial properties. Based on these findings, the fungal isolates are proven to be useful as potential biological control agents against *E. mallotivora,* and the genomic data opens possibilities to further explore the underlying molecular mechanisms behind their antimicrobial activity, with potential synthetic biology applications.

## 1. Introduction

Papaya dieback disease (PDD), or bacterial crown rot (BCR), is a bacterial-caused disease that has negatively affected papaya crops (*Carica papaya* L.) in Malaysia, reducing the national papaya export up to 70% [1]. Initially, *Erwinia papayae* was reported to be the causal agent of PDD [2], though later Mat Amin et al. [3] molecularly validated the pathogen to have the closest match to *E. mallotivora* Goto strain DSM 4565 and this was further supported through genome sequencing [4]. Most of the Malaysian papaya cultivated varieties (cultivars) such as ‘Eksotika’, ‘Sekaki’, and ‘Setiawan’ are highly susceptible to this Gram-negative pathogen [5]. Once infected, water soak symptoms become prevalent on papaya petioles, leaves, and the apical stem, and the tree can succumb to the disease within one to two weeks [5,6,7]. In addition to physical diagnosis, Mohd Said et al. [8] developed a DNA-based detection method for *E. mallotivora* to further assist in early detection of the disease and Ramachandran et al. [9] conducted a repetitive element PCR fingerprinting (rep-PCR) study to provide an accurate and rapid identification method on various local *E. mallotivora* isolates. Currently, there is no effective cure for PDD from the moment the symptoms appear on the trees. Hence, farmers rely on preventive measures and good management practices to contain and minimise the spread of the disease to neighbouring trees and plots [5,10,11]. Recently, *E. mallotivora* was reported to be affecting papaya in the Philippines and Indonesia, making this species a pathogen of concern that could affect the papaya industry in the entire Southeast Asia region [7,12]. A similar disease termed as papaya black rot has been recently detected in Japan and the pathogen was characterised to be from the genus *Erwinia* [13].

Numerous studies have been conducted in recent years to understand the pathogenesis of *E. mallotivora* and find solutions to curb the disease. About four years after the identification of the pathogen, the draft genome sequence of *E. mallotivora* was published and this has initiated more research [4]. Wee et al. [14], Hamid et al. [15], Juri et al. [16,17], Abu-Bakar et al. [18], Abu Bakar et al. [19], and Tamizi et al. [20] have conducted in-depth studies to understand the host–pathogen interactions and pathogenicity of *E. mallotivora* at the molecular level, while Noor Shahida et al. [6] described the infection from a physiological perspective. The use of conventional breeding and genetic engineering to develop new papaya lines that would be resistant to *E. mallotivora* is still in the research pipelines [21,22,23,24,25]. Nevertheless, the effort to combat the disease should be augmented with other approaches, including the use of effective microorganisms and native microbial biocontrol.

Mat Amin et al. [26,27] discovered several *Bacillus* spp. from soil to be antagonistic towards *E. mallotivora*, and this effort has pioneered the search for potential antagonistic microbes against *E. mallotivora* that have been ravaging papaya trees. Later, a biocontrol inoculant containing an undisclosed species of *Bacillus* sp. known as ‘Dieback Buster 95′ was launched and claimed to be highly efficient in the prevention of PDD [28,29]. In addition to these, native endophytic bacteria (NEB) isolated from papaya were reported to be capable of suppressing the growth of *E. mallotivora* [30,31].

While these potential biocontrols have been isolated from the kingdom of bacteria, fungal species possessing antagonistic effects against *E. mallotivora* remain unexplored. Fungi of the genus *Trichoderma* have been widely studied for their biological control properties against pathogenic fungi and other bacterial species [32,33,34,35]. They are known plant growth-promoting fungi (PGPF), abundant in soil and easily culturable [36,37]. According to Guo et al. [37] and Khan et al. [38], *Trichoderma* species produced diverse compounds and active metabolites during their interaction with competitors. This makes an effort to single out a potential antimicrobial compound from a particular *Trichoderma* species relatively challenging. Rush et al. [39] produced a systemic review on *Trichoderma* bioprospecting which discussed the application of genome technology in screening for natural products and biocontrol compounds. In the present study, we have discovered five *Trichoderma* isolates that showed antimicrobial activity against the *E. mallotivora* strain BT-MARDI, of which three were further characterised through genome sequencing to investigate the possible mechanisms behind their antimicrobial properties.

## 2. Materials and Methods

### 2.1. Preparation of Pathogen

The papaya dieback pathogen, *E. mallotivora* strain BT-MARDI, was obtained from the Biotechnology and Nanotechnology Research Centre, at the Malaysian Agricultural Research and Development Institute (MARDI) in Serdang, Selangor, Malaysia. A stock culture of *E. mallotivora* was maintained in 15% (*w*/*v*) glycerol at −80 °C. Working cultures were established by streaking the glycerol stock onto Luria Bertani (LB) agar plates (10 g/L tryptone, 5 g/L yeast extract, 10 g/L sodium chloride, 15 g/L agar) and incubating at 30 °C for 48 h. Fresh single colonies were then picked and cultured in 50 mL LB medium (10 g/L tryptone, 5 g/L yeast extract, 10 g/L sodium chloride) in a 250 mL flask, shaking at 180 rpm for 24 h.

### 2.2. Isolation of Antagonist Fungi

The rhizosphere soil samples were randomly collected from healthy papaya plants (wild and cultivated) at five different locations (Table 1) in Peninsular Malaysia. Then, soil dilution was performed and used to grow culturable fungi, particularly *Trichoderma*, on potato dextrose agar (PDA) supplemented with selective agents. Briefly, 50 g of soil sample were diluted in 50 mL of sterile distilled water. Serial dilution was performed at 1/10, 1/100, 1/1000, and 1/10,000. Four hundred microliters of the diluted soil suspension were spread on modified PDA (PDA; 20 g/L glucose, 4 g/L potato extract, 20 g/L agar) medium containing 0.3 g/L chloramphenicol, 0.1 g/L streptomycin, and 0.02 g/L Rose Bengal dye [40]. The plates were incubated at 25 °C for 3 to 14 days to allow fast and medium–fast growing fungi to appear. The emerging fungal colonies were then purified by single spore isolation [41] and maintained on PDA slants at 4 °C. Long-term storage of fungi was achieved by cutting a 5 mm^2^ of mycelial agar plug from freshly growing cultures, which was stored in 30% (*w*/*v*) glycerol at −80 °C.

### 2.3. Antagonistic Activity of Fungal Isolates against E. mallotivora Strain BT-MARDI

The antagonistic activity of fungal isolates against *E. mallotivora* was screened in vitro using the agar well diffusion method on PDA plates [31,42,43]. An agar plug (5 mm^2^) from each five-day old fungal culture was inoculated into 100 mL potato dextrose broth (PDB; 20 g/L glucose, 4 g/L potato extract) in 500 mL flasks and grown for seven days at 25 °C with agitation at 180 rpm. A lawn of *E. mallotivora* was prepared by spreading 100 µL of the *E. mallotivora* inoculum over the entire agar surface. A hole with a diameter of 6 mm was punched aseptically with a sterile cork borer and 100 µL of the fungal suspension were dispensed into the well. A kanamycin solution (100 μg/mL) and PDB, 100 μL of each, were used as the positive and negative controls, respectively. After overnight incubation at 28 °C, the plates were observed for zone of inhibition formation on the *E. mallotivora* lawn. The experiment was repeated in triplicate for each isolate to record the average diameter of the inhibition zone. The data for fungal isolates exhibiting significant antagonism compared to the positive control was analysed using IBM SPSS Statistics version 28.0.1.1 (14).

### 2.4. Genomic DNA Extraction

Cultures of the three selected fungal isolates, UKM-M-UW RA3a, UKM-M-UW RA5, and UKM-M-UW RA6 (hereafter referred to as RA3a, RA5, and RA6), were set up in 25 mL of PDB from PDA plates and placed in 250 mL conical flasks. The fungal cultures were incubated at 25 °C for 72 h, shaking at 200 rpm. The mycelia from the broth were pelleted by centrifugation at 8000× *g* for 5 min, and subsequently washed twice with sterile distilled water to remove any residual medium. High molecular weight (HMW) genomic DNA extraction was carried out via cryogenic grinding using a sterile mortar and pestle. Fungal mycelia were ground into a fine powder in liquid nitrogen; thereafter, 200 mg of the powder were transferred aseptically into a microcentrifuge tube for immediate use. A GenElute Plant Genomic DNA miniprep kit (Sigma-Aldrich, Burlington, MA, USA) was used for DNA extraction, following the manufacturer’s instructions. HMW genomic DNA was concentrated using ethanol precipitation. Briefly, 1/10 volume of 3 M sodium acetate, pH 5.2, was added, followed by three volumes of 100% ethanol. Samples were inverted, incubated at −20 °C overnight, then spun at 13,000× *g* for 30 min at 4 °C. Supernatant was decanted, the DNA pellet was air dried, and then resuspended in nuclease-free water (Thermo Fisher, Waltham, MA, USA). Gel electrophoresis (1% *w*/*v* agarose gel with 1X GelRed), a Nanodrop ND1000 (Thermo Fisher, USA), and a Qubit (dsDNA Broad range, Invitrogen, Waltham, MA, USA) were used to assess the concentration (Table S1) and quality of the HMW genomic DNA extracted from the fungal strains. 

### 2.5. Identification of Fungi Based on Mycelia Morphology, PCR Amplification, and Sanger Sequencing 

Mycelial morphology was originally used to identify the class/genus of the fungal isolates based on the colour of the mycelia and spore characteristics. Subsequently, PCR amplification of target sequences was carried out using purified genomic DNA as a template to identify the fungal isolates of interest, RA3a, RA5, and RA6. PCR was carried out using Q5 High-Fidelity 2X Master Mix (NEB, Ipswich, MA, USA) according to the manufacturer’s instructions, using the primers listed in Table S2. The amplicons were purified upon gel electrophoresis (1% *w*/*v* agarose gel with 1X GelRed) using the GeneJET PCR purification kit (Thermo Fisher, USA) following the manufacturer’s instructions. One hundred nanograms of the purified PCR product was used for Sanger sequencing through Eurofins-GATC, which enabled for the phylogenetic confirmation of the strains. The sequences were trimmed to obtain diagnostic fragments for molecular identification as detailed in Kopchinskiy et al. [44] prior to submission to BLASTN [45].

### 2.6. DNA Library Preparation and Sequencing through Oxford Nanopore Technology (ONT)

Utilising the extracted HMW genomic DNA, preparation of the library was performed using the native barcoding expansion kit (EXP-NBD104 and EXP-NBD114) and the ligation sequencing kit (SQK-LSK109) for multiplex genomic DNA sequencing of the fungal isolates RA3a, RA5, and RA6. NBD01 was used to tag RA3a, NBD02 for RA6, and NBD03 for RA5. The manufacturer’s protocol was followed with minor modifications introduced for both DNA repair and end-prep stages, by increasing the incubation time and temperature after washing with ethanol to 15 min at 37 °C. Furthermore, at the native barcode ligation step, the incubation time and temperature were increased to 30 min at 37 °C. To enrich DNA fragments of 3kb or longer, the Long Fragment Buffer was used for the DNA clean-up. An amount of 1.2 µg of each genomic DNA were used for the library preparation, resulting in a final amount of 350 ng of DNA at the end of the adapter ligation and clean-up step. The resulting DNA library containing the sequencing buffer and loading beads were loaded on the primed SpotON flow cell. Nanopore sequencing was performed on MinION (ONT) with a FLO-MIN-106 R9.4 flow-cell (ONT). The MinION (ONT) multiplex sequencing reaction was run for 18 h.

### 2.7. Genome Assembly and Error Corrections

A *de novo* strategy was employed for sequencing each of the three genomes. The raw data produced by nanopore was base-called and simultaneously demultiplexed using Guppy version 4.4.1 available to ONT users via https://community.nanoporetech.com (accessed on 23 January 2022). The config file used in Guppy was dna_r9.4.1_450bps_hac.cfg, the remaining parameters used were the default settings. Guppy was also used to trim the barcodes from the reads during the base-calling, depositing each genome’s reads in a directory as .fastq files. The .fastq files for each read were merged into one .fastq file and used as the input for Flye version 2.8.2 with the nano-raw mode selected, which was used to assemble the draft genomes [46]. The draft assembly was then polished multiple times, by aligning the base-called raw reads in the merged file against the draft genome using Minimap2 version 2.11 [47] before correcting errors using Racon version 1.4.20 [48], where matching bases were assigned a score of 8 and mismatches a score of −6, with a gap penalty of −8 and a window size of 500. The output from Racon was aligned to the raw reads again and the process was repeated three times for each assembly. Further corrections were made using Medaka version 1.2.1 utilising model r941_min_high_g360. 

Polishing the genome continued by using Illumina short-read data generated through Apical Scientific Sdn. Bhd. (Seri Kembangan, Selangor, Malaysia). This was done by aligning the Illumina data to the output from Medaka using Bowtie2 version 2.4.2 [49]. Bowtie2 was run with the following settings, -D (number of attempts at extension before skipping to the next task) 20, -R (number of seeds looked at before moving to the next task) 3, -N (maximum number of mismatches in alignments) 1, -L (length of seed substrings) 20, and -i (interval between seed substrings) S,1,0.50, all other settings were left at the default parameters. Polishing was performed with Pilon version 1.23 [50], the output from Pilon was then re-aligned to the Illumina reads and the draft genome polished again; after Pilon had been run four times, the final output was analysed using BUSCO version 5.0.0 [51], comparing the three genomes against the Hypocreales_odb10 database. Unless specified above, parameters within programs were left at their default setting.

### 2.8. Genome Annotations

Functional annotation was performed using the Funannotate version 1.8.3 pipeline [52]. Funannotate was also able to clean (remove small repetitive contigs by using minimap2 or mummer) and perform mask repeats (softmasking of low complexity and short period tandem repeats using tantan). The prediction of functional elements was done using a seed species of *Verticillium longisporum* and the BUSCO *Sordariomycetes* database; the minimum number of training models required was dropped to 100, and the Optimize Augustus setting was turned on. Funannotate predict uses AUGUSTUS, snap, GlimmerHMM, and tRNAscan-SE in order to predict genes for proteins and tRNAs, as well as other programs to generate the inputs for those mentioned above and the final outputs. The predictions were then run through Phobius [53] and AntiSMASH [54], contained within Funannotate. Finally, the annotation function was used to create the final genomes. BUSCO was run again to look at the predicted proteins generated, once again using the Hypocreales_odb10 database. All parameters not mentioned above were left on their default setting within the software package.

CMscan [55] was used with StructRNAfinder [56] for screening the presence of potential non-coding RNA. The Rfam database [57] was used as the input database for StructRNAfinder with the default parameters.

### 2.9. Comparative Genomics and Phylogenomic Analysis

Pairwise genomic similarities between our isolates with addition of nine additional genomes from other *Trichoderma* spp. (obtained from the NCBI database) were calculated using FastANI, with assembled genomic sequences as input [58]. The pairwise genomic similarities were then visualised using the Intervene Shiny package [59]. Next, to reconstruct the phylogenomic association between several strains of *Trichoderma* spp., single-copy orthologous proteins were first identified from the predicted proteome sequence via OrthoFinder v2.2.7 with default settings [60]. Then, amino acid sequences from single-copy orthologous proteins identified were aligned using MAFFT [61]. This alignment was then used for maximum likelihood phylogenomic tree reconstruction via the IQ-TREE program, using the JTT+F+G4 model and 1000 ultrafast bootstrap replications [62]. iTOL was used for phylogenomic tree visualisation [63]. Finally, OrthoVenn2 web server was used for comparison of orthologous proteins from our isolates with the predicted proteome as input [64].

## 3. Results

### 3.1. Antagonism of Fungal Isolates against E. mallotivora Strain BT-MARDI

**Figure 1:**
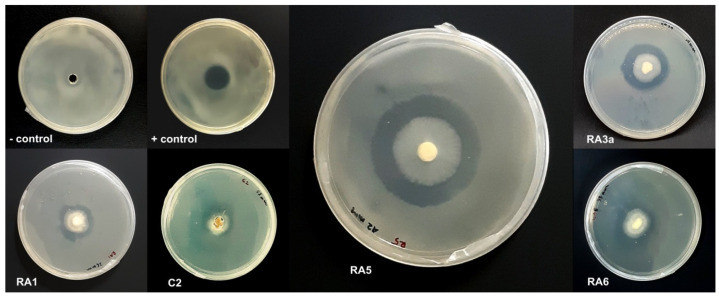
Plate-based growth inhibition assays of *E. mallotivora* through well diffusion method. All fungal isolates displayed notable growth inhibition zones. Potato dextrose broth (PDB) and kanamycin were used as the negative and positive controls, respectively.

**Figure 2:**
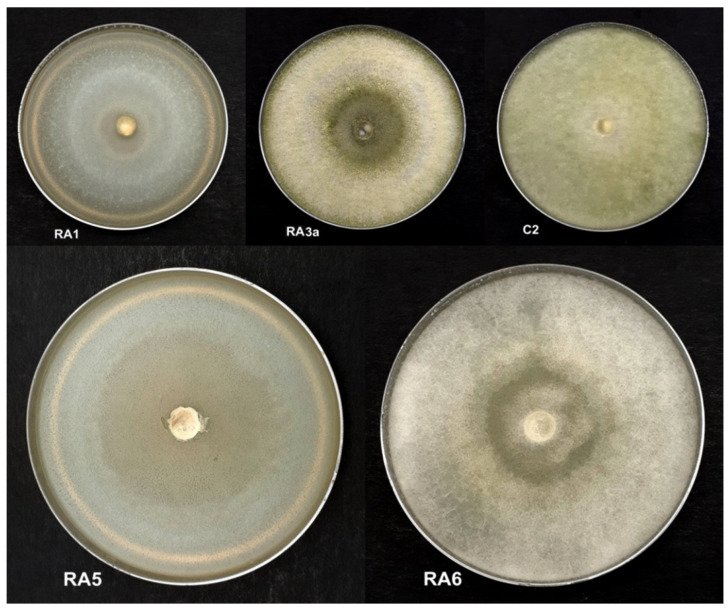
Physical morphology of the five fungal isolates RA1, RA3a, C2, RA5, and RA6 cultured on PDA plates growing filamentous mycelia across the agar after five days of incubation. All isolates show the morphological characteristics of *Trichoderma*—white filaments and, as the isolates mature, faint olive-green spores.

**Figure 3:**
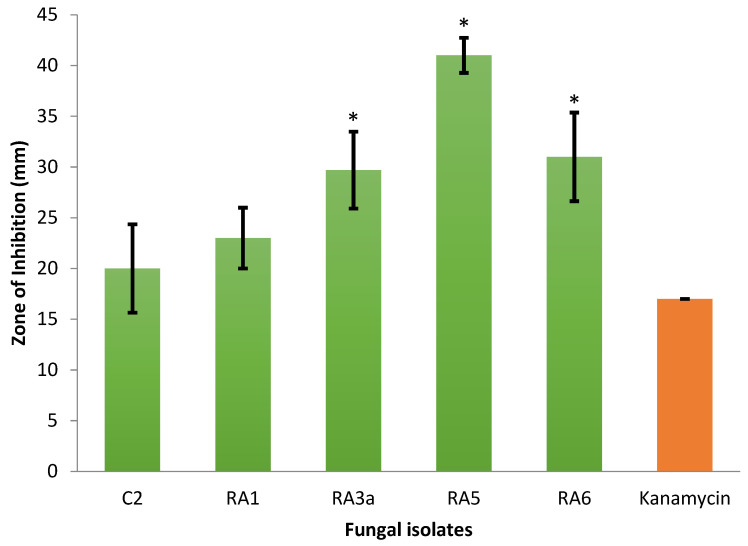
In vitro screening for antagonism of five fast-growing fungal isolates (*Trichoderma* spp.) against *E. mallotivora* ‘BT-MARDI’ using the agar well diffusion method. Values are means of triplicates and the standard deviations are indicated by the error bars. Isolates with significant antagonism (compared to kanamycin) were determined using the Tukey’s test (N = 18, *p* < 0.05) and are marked with an asterisk (*).

In this study, a total of 128 filamentous fungal and yeast isolates were cultured from five soil samples collected in various locations in Peninsular Malaysia (Table 1). Out of these, only 17 fungal isolates that grew mycelia in less than 14 days on PDA plates—species putatively belonging mainly to the genera *Trichoderma*, *Fusarium*, *Aspergillus,* and *Penicillium* based on morphological identification—were observed to have antagonistic activity against *E. mallotivora* strain BT-MARDI. According to plate-based inhibition assays, five fast-growing fungal isolates that required less than seven days to form a full mycelial lawn on PDA plates were confirmed to exhibit visible antagonism (Figure 1) and selected for further analysis. The taxonomic affiliation of these isolates was originally deduced based on mycelium morphology, with all five strains, or isolates, putatively assigned to the genus *Trichoderma* (Figure 2). All isolates exhibited inhibition of the growth of *E. mallotivora* (Table 2). Based on the Tukey’s test, RA5, RA6, and RA3a had a significant antagonistic activity when compared to the positive control (kanamycin) (Figure 3). 

### 3.2. Genomic DNA Extraction

High molecular weight (HMW) genomic DNA extraction was performed on the three fungal isolates, RA3a, RA5, and RA6, that showed significant inhibition on *E. mallotivora*. To improve the genomic DNA yield and quality, the extracted fungal DNA was subjected to ethanol precipitation and resuspended in 35 µL of nuclease-free water. The purity and concentration of the DNA was assessed with a Nanodrop ND1000 (Thermo Fisher, Boston, MA, USA) and Qubit (dsDNA Broad range, Invitrogen, Boston, MA, USA), with all preparations yielding more than 100 ng/µL HMW DNA according to Qubit readings (Table S1). The HMW DNA was further visualised via agarose gel electrophoresis (Figure S1). Overall, these analyses proved that the HMW DNA extracted from the three isolates was suitable to be sequenced through Oxford nanopore and Illumina whole-genome sequencing.

### 3.3. Molecular Identification of Strains

The three fungal isolates RA3a, RA5, and RA6 were identified through PCR amplification and Sanger sequencing of three DNA barcodes [65]: internal transcribed spacer (ITS) [66], translation elongation factor 1 alpha (*tef1*) gene [67] and RNA polymerase B subunit II (*rpb2*) gene [68]. Amplification of each sample yielded a single DNA fragment, the sequence of which was analysed through BLASTN [45]. The sequencing of the ITS marker allowed us to confidently assign all three fungal isolates to the *Trichoderma* genus. Further sequencing of the *tef1* and *rpb2* genes enabled us to identify all three fungal strains to be from a single species, *T. koningiopsis* (Table 3).

### 3.4. Genome Sequencing

The genomic DNA of the three fungal isolates, RA3a, RA5, and RA6, were sequenced using a combination of Oxford nanopore and Illumina whole-genome sequencing. This generated a significant quantity of both long-read and short-read data which gave a high degree of coverage for each of the three genomes. From the nanopore data, after barcodes had been separated and trimmed, we had generated an estimated 3,176,623,257 bases for strain RA3a, 3,973,025,475 bases for RA5, and 2,406,065,838 bases for RA6. This was complemented by the Illumina sequencing which produced 3,352,912,241 bases for RA3a, and 3,365,209,722 and 2,697,750,185 for RA5 and RA6, respectively. The final draft genome sizes for each organism were as follows: RA3a 36.53 Mb, RA5 36.48 Mb, and RA6 36.47 Mb. This is consistent with the size of other *Trichoderma* genome assemblies which tend to be 31–40 Mb in size [69]. As shown in Table 4, the genomes contain 14 (RA3a), 11 (RA5), and 13 (RA6) contigs, the majority of these contigs may be putatively assembled at the chromosome level. Closely related species of *Trichoderma* have seven chromosomes [70], and our assemblies also included the mitochondrial DNA.

Using BUSCO [71] to assess the genome assemblies for core conserved genes across the order Hypocreales, we were able to generate the results presented in Table 5 and Table 6. Table 5 shows the conserved genes identified within the scaffold of our assemblies and Table 6 shows the conserved proteins identified in the predicted proteome of the annotated genomes of each species. An outline of the predicted non-coding RNA is shown in Table S3, the full set of results from StructRNAfinder are publicly available at https://osf.io/vsbc2/ (Accessed on 17 February 2022).

### 3.5. Genome Analysis

While the gene function of the predicted genes in each of the three genomes was assigned using Funannotate, the predicted proteome of each strain was run again through Eggnog-mapper v2.1.4-2. This yielded the following results, where for strain RA3a of the 8,951 predicted proteins, 86.7% could have a COG (Clusters of Orthologous Groups) category assigned; similarly, of the 8964 proteins predicted in strain RA5, 86.7% could have COG categories assigned, and 86.2% of the 9124 predicted proteins from RA6 could be too. 

**Figure 4:**
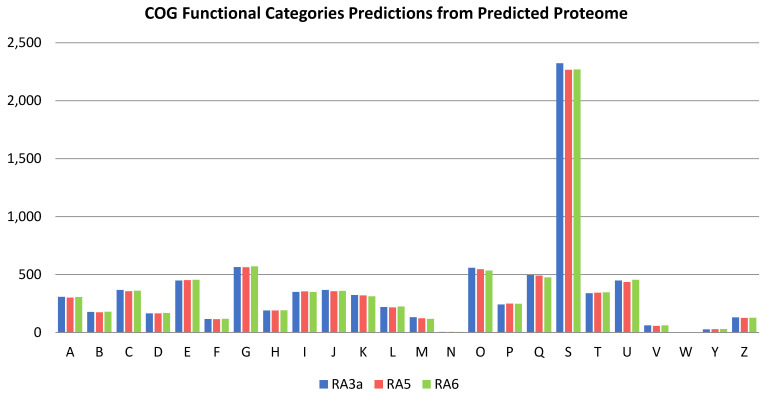
Distribution of predicted proteins in the three fungal isolates RA3a, RA5, and RA6 across the different COG categories. A: RNA processing and modification. B: Chromatin structure and dynamics. C: Energy production and conversion. D: Cell cycle control, cell division, chromosome partitioning. E: Amino acid transport and metabolism. F: Nucleotide transport and metabolism. G: Carbohydrate transport and metabolism. H: Coenzyme transport and metabolism. I: Lipid transport and metabolism. J: Translation, ribosomal structure. and biogenesis. K: Transcription. L: Replication, recombination. and repair. M: Cell wall/membrane/envelope biogenesis. N: Cell motility. O: Posttranslational modification, protein turnover, chaperones. P: Inorganic ion transport and metabolism. Q: Secondary metabolites biosynthesis, transport. and catabolism. S: Function unknown. T: Signal transduction mechanisms. U: Intracellular trafficking, secretion, and vesicular transport. V: Defense mechanisms. W: Extracellular structures. Y: Nuclear structure. Z: Cytoskeleton. The following returned no hits: R: General function prediction only; X: Mobilome- prophages, transposons.

The distribution of these predicted proteins across the COG categories is shown in Figure 4. Distribution of proteins across the categories did not differ significantly across any of the three genomes, and while the most frequently mapped category was “Function Unknown”, of those that could be placed into a category of known function, the five most common in order of decreasing predicted protein count within the categories were “Intracellular trafficking, secretion, and vesicular transport”, “Amino acid transport and metabolism”, “Secondary metabolite biosynthesis, transport and catabolism”, “Posttranslational modification, protein turnover, chaperones”, and “Carbohydrate transport and metabolism”. The presence of over 475 proteins associated with secondary metabolites in each genome is promising and these deserve further examinations, particularly in the context of the antagonistic activity of the fungal isolates against *E. mallotivora* and, potentially, other microorganisms. It is also interesting that no mobilome elements associated with transposons and prophages were detected, and less than 100 predicted proteins were identified in each genome that were associated with any of the following categories: “Extracellular structures”, “Cell motility”, “Nuclear structure”, and “Defence mechanisms”.

**Figure 5:**
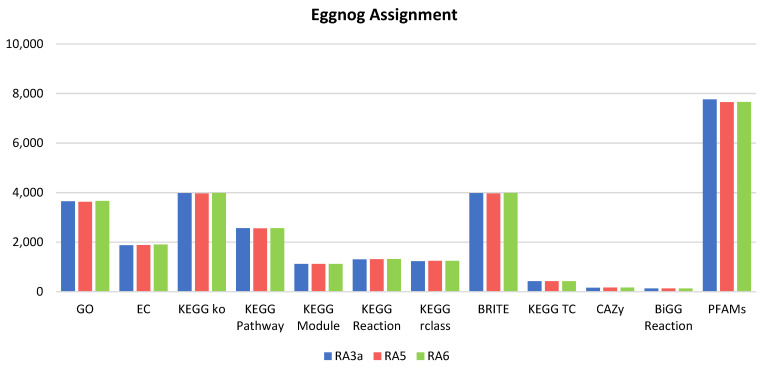
Assignment of predicted proteins in the three fungal isolates RA3a, RA5, and RA6 using eggnog mapper to multiple databases.

Figure 5 shows that the eggnog mapper was also able to assign gene ontology terms; often multiple terms are assigned to individual proteins, as well as enzyme commission numbers, a variety of matches to multiple KEGG databases, and also to BRITE hierarchies. A limited number of predicted proteins (no more than 2% in any genomes) were also associated with matches to the CAZy database of carbohydrate active enzymes, as well as matches to BiGG IDs. More promising was that around 85% of genes in each genome were matched to proteins in the pfam database. There was considerable overlap between these categories, however; most genes are close to identical across each of the three genomes. All data generated using eggnog mapper are included in the Dataset S1.

### 3.6. Comparative Genomics and Phylogenomic Analysis

**Figure 6:**
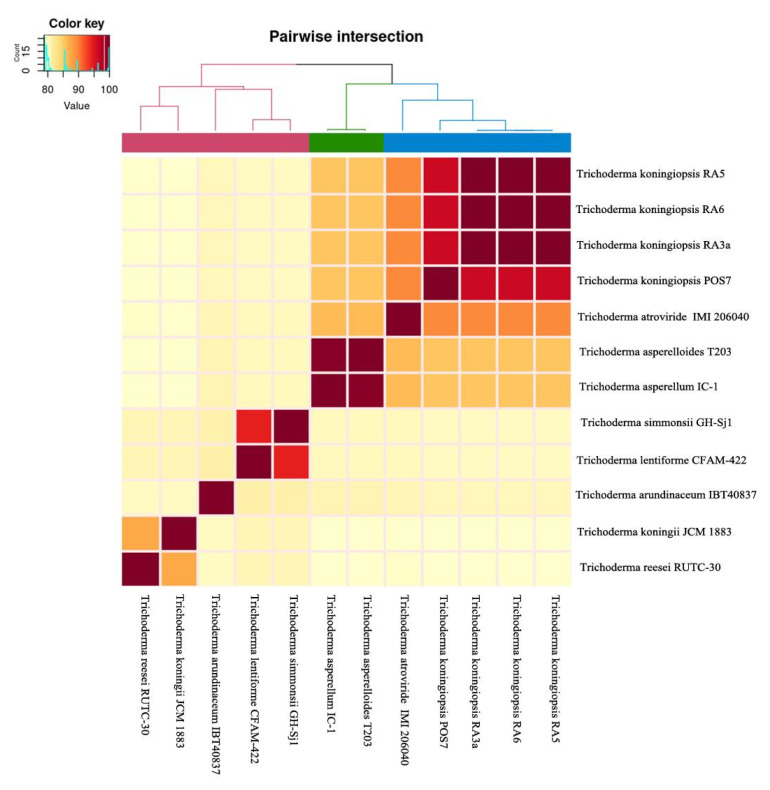
The ANI values based on the FastANI algorithm for *Trichoderma* spp. related genomes. The clustering was done based on a Euclidean distance matrix.

Pairwise comparison of genomic similarities between our isolates was performed by calculating the average nucleotide identity (ANI) values. The three isolates, RA3a, RA5, and RA6, showed high genomic similarities between each other (ANI value of 99%) in addition to the *T. koningiopsis* POS7 isolate, which shared an ANI value of 96% (Figure 6). Conversely, the three isolates showed lower genomic similarities with other *Trichoderma* spp. (79–89% of ANI value). These results further confirm the identity of our isolates as belonging to the *T. koningiopsis* species.

Further analysis using OrthoFinder suggested that there is a total of 12,270 orthogroups present among the predicted proteomes used in our analysis. Among that, a total of 3392 of the orthologous proteins exist as single-copy and alignment of their amino acids sequence was used for phylogenomic tree construction. The phylogenomic tree generated shows that the three isolates formed a distinct monophyletic group and shared a recent common ancestor within *T. atroviride* IMI206040 (Figure S2). The robustness of the phylogenomic tree generated was confirmed as all the branches showed 100% bootstrap values.

Next, the predicted protein sequences from our isolates were compared with each other using the OrthoVenn2 web server. All the predicted protein sequences extracted from our genomes were further grouped into 9225 orthologous proteins. Among these, a total of 8245 protein groups were shared by all isolates. These proteins accounted for 86.64 (RA3a), 89.09 (RA5), and 89.00% (RA6) of the total orthologous protein group encoded in each genome (Figure S3). Therefore, OrthoVenn2 analysis confirmed that most of the proteins encoded for by the three isolates are shared. 

### 3.7. Secondary Metabolite Clusters

**Figure 7:**
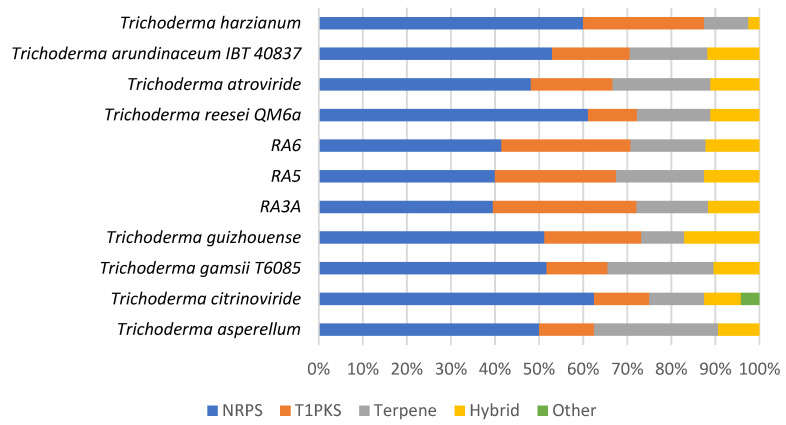
Comparison of the BGCs found in the genomes of the fungal strains RA3a, RA5, and RA6 with other members of the *Trichoderma* genus. BGC prediction was performed through AntiSMASH.

The whole genome sequences of the *Trichoderma* isolates RA3a, RA5, and RA6 were mined for putative biosynthetic gene clusters (BGCs) using AntiSMASH [54]. A total number of 124 clusters were identified across the three genomes; 43 BGCs were identified in isolate RA3a, 40 in isolate RA5, and 41 BGCs in isolate RA6. The strains varied in the putative classes they encode (Table 7 and Dataset S2). Non-ribosomal peptide synthetase (NRPS) clusters are the most dominant across the isolates followed by polyketide synthase (PKS) clusters, and Terpene and hybrid NRPS-PKS clusters. The number of BGCs from the sequenced fungal strains (RA3a, RA5, and RA6) was compared to the number of BGCs from other members of the *Trichoderma* genus (Figure 7), as predicted by AntiSMASH. This revealed that the fungal strains isolated in this study have the potential to be talented producers of polyketides and non-ribosomal peptide synthases, in line with other members of the *Trichoderma* genus.

We then screened for clusters that were consistently present across the three strains, which could be indicative of their shared bioactivity against *E. mallotivora*. As predicted by AntiSMASH, we could find BGCs with low-to-high similarity to those for known bioactive metabolites, namely fusaric acid, naphthopyrone, neurosporin A, ascochlorin, and clavaric acid (Table S4 and Dataset S3). Further manual BlastP analysis of the putative neurosporin A BGC, revealed that this cluster may in fact code for the biosynthesis of a salicylaldehyde-related compound. This observation is based on the identification within the BGC of a hr-PKS megasynthase and tailoring enzymes, all showing high homology with genes of the *vir* gene cluster, which is involved in the biosynthesis of the antimicrobial compounds trichoxide and virensols from the biocontrol fungus *T. virens* [72] (Table S5 and Figure S4). 

## 4. Discussion

*Trichoderma* spp. are easily culturable and generally considered to be harmless to humans and animals—except for *T. longibrachiatum* [73,74]. Members of this genus have been used extensively in agriculture as eco-friendly biological control agents as they are capable to control (or suppress) other microorganisms directly or indirectly [37,38,75,76]. It is reported that various *Trichoderma* spp. are effective biocontrol means against many fungal pathogens and some bacterial pathogens [77]. Previous works explored and applied the idea of using microbial species to control *E. mallotivora* [26,27,28,29,30,31]; nevertheless, the use of *Trichoderma* as a fungal antagonist against the PDD pathogen has not been reported. In this study, we demonstrated the potential of five *Trichoderma* strains to cause growth inhibition of *E. mallotivora* strain BT-MARDI. *Trichoderma* offers an advantage over many other microbes (especially bacteria) in terms of fast rhizosphere colonisation, involvement in the soil nutrient cycle, and excellent viability after an extended storage period (>12 months), thus making the fungus more efficient and appealing to the farmers [78,79]. In addition to their antagonistic features against pathogens, some species of *Trichoderma* are even capable of inducing plant defence mechanisms, which could be another important advantage [80].

To further gain insights on the antagonism of our isolates, we performed whole-genome sequencing on three fast-growing *Trichoderma* strains that showed significant inhibition of *E. mallotivora* to aid in the elucidation of the metabolites that might play a role in this interaction. From the sequencing results, we believe that the annotated draft genomes we produced can be treated as full open reading frames. Mutations and evolutionary divergence can explain some of the fragmented BUSCO scores, and even well-conserved genes can still be lost in some lineages [81]. The large coverage of the long read nanopore sequencing allowed for a robust first draft genome to be created, with few fragments. However, nanopore read base calling remains less accurate than Illumina, around 95% [82], and while modern base calling software harnessing neural networks are improving this, it is still necessary to polish, with higher accuracy, short read Illumina sequencing data to create a robust genome assembly, in order to remove the indels and miscalled bases present in nanopore reads that otherwise lead to frameshifts and fragmentation of genes.

The genus *Trichoderma* is a well-recognised group of filamentous fungi known for their production of secondary metabolites, especially as talented producers of bioactive peptides, polyketides, plant growth regulators, enzymes, siderophores, and other antibiotics [38,81,83], and has been credited for its biocontrol activity as antifungal and antibacterial. For example, peptaibols are a well-studied class of peptide natural products from *Trichoderma,* synthesised by NRPS modules, producing a linear peptide consisting of dialkylated amino acids, isovaline, amino isobutyric acid (Aib), an acetylated N-terminus, and a C-terminal amino alcohol [38]. They are credited for their antimicrobial properties, as well as their ability to induce systemic resistance in plants against microbial invasion. Another major class of *Trichoderma* natural products with biocontrol activity are koninginins. This family of compounds were first isolated from *T. koningii* and exhibited antifungal activity [38,84]. Interestingly, koninginins have also been isolated from *T. koningiopsis* in other studies and are reported to exhibit antifungal properties against *Fusarium* spp., *Plectosphaerella cucumerina,* and *Alternaria panax* [85]. Prominent examples of polyketides isolated from *Trichoderma* spp. include pyrones and pyridines [38,86]. Considering the diversity of bioactive molecules isolated from the genus—for a report about *Trichoderma* natural products we refer the reader to the review by Shenouda and Cox [86]—and given the vast biosynthetic potential emerged from AntiSMASH analysis conducted in our study, the three *Trichoderma* strains (RA3a, RA5, and RA6) have high potential to produce bioactive molecules that will warrant their use as biocontrol agents against plant pathogens. Noteworthy, we have identified BGCs conserved across the three fungal isolates, including one for the biosynthesis of putative salicylaldehyde-related compounds, which are known for their antimicrobial properties, such as those that have been isolated from *T. virens* [72] and *T. citrinum* [87]. Heterologous expression and/or knockout of the salicylaldehyde BGC homologues from RA3a, RA5, and RA6 will be needed to reveal the structure of the corresponding metabolites and their potential role in the bioactivity of the fungal isolates.

The dogma of biosynthetic studies remains that many fungal biosynthetic clusters are silent under standard laboratory conditions, which makes their full exploitation challenging [88,89]. Advances in synthetic biology via heterologous expression or genome editing may help us uncover the biosynthetic potential of the gene clusters [90,91].

## 5. Conclusions

In this study, we have isolated fungal strains from soil samples collected in the rhizosphere of healthy papaya trees from different locations in Peninsular Malaysia, with the aim to identify a potential biological control agent capable of suppressing *E. mallotivora*, the pathogen that is responsible for the ongoing PDD outbreak in Malaysia and surrounding countries. The three *Trichoderma* isolates, UKM-M-UW RA3a, UKM-M-UW RA5, and UKM-M-UW RA6, have shown significant inhibition of the growth of *E. mallotivora* from plate-based bioassays, and molecular identification allowed us to assign them to the *T. koningiopsis* species. Whole-genome sequencing was performed, thereby providing a platform for their biosynthetic exploitation, with the goal of linking secondary metabolites to biosynthetic gene clusters. Biosynthetic gene clusters homologous to those for known bioactive metabolites were identified and found to be conserved across the three isolates, opening the way for future exploration of the biosynthetic potential of these fungi. With the growing need for greener alternatives to chemical pesticides, the biosynthetic studies on natural products from *Trichoderma* spp. is expected to grow, which may give rise to a new generation of biocontrol agents with an enormous impact in the agrochemical sector.

## Figures and Tables

**Table 1:** Origin of the soil samples used for the isolation of the fungi identified in this study. The soil samples were collected from wild and cultivated healthy papaya populations in different states (Selangor, Negeri Sembilan, Pahang, and Kedah) of Peninsular Malaysia.

Soil Sample ID	Location Site, State	Coordinates
Mardi-2018-TK	Tanjong Karang, Selangor	3°26′27.8″ N 101°08′50.1″ E
Mardi-2018-R	Rembau, Negeri Sembilan	2°34′26.0″ N 102°05′48.5″ E
Mardi-2018-P	Raub, Pahang	3°49′19.3″ N 101°50′38.4″ E
Mardi-2018-C	Mardi Serdang, Selangor	2°59′24.9″ N 101°41′49.5″ E
Mardi-2018-BK	Baling, Kedah	5°40′05.6″ N 100°49′47.5″ E

**Table 2:** Antagonistic potential of the *Trichoderma* isolates against *E. mallotivora.* The negative (−) control consists of 100 μL of PDB, the positive (+) control of 100 μL of kanamycin (100 μg/mL).

Treatment	Diameter of Inhibition Zone (mm)					Fungal Genus (Based on Morphology)	Origin of the Fungal Isolate
	Replicate			Mean	Standard Deviation		
	1	2	3				
− control	0	0	0	0	0	-	-
+ control	17	17	17	17.0	0	-	-
Isolate UKM-M-UW RA5	42	39	42	41.0	1.73	*Trichoderma*	Rembau, Negeri Sembilan
Isolate UKM-M-UW RA6	33	26	34	31.0	4.36	*Trichoderma*	Rembau, Negeri Sembilan
Isolate UKM-M-UW RA3a	34	28	27	29.7	3.79	*Trichoderma*	Rembau, Negeri Sembilan
Isolate UKM-M-UW RA1	20	23	26	23.0	3.0	*Trichoderma*	Rembau, Negeri Sembilan
Isolate UKM-M-UW C2	15	22	23	20.0	4.36	*Trichoderma*	Serdang, Selangor

**Table 3:** Molecular identification of the three fungal isolates RA3a, RA5 and RA6 based on Sanger sequencing of the three DNA barcodes ITS, *Tef1* and *Rpb2*.

Isolate	Locus	Closest Match Organism	NCBI Accession Number	Coverage (%)	Identity (%)
UKM-M-UW RA3a	ITS	*Trichoderma sp.* strain ZMQRS9	MT446202.1	100	99.83
	*Tef1*	*Trichoderma koningiopsis* strain LESF360	KT278986.1	100	99.65
	*Rpb2*	*Trichoderma koningiopsis* isolate Tkois1	MT081443.1	100	99.77
UKM-M-UW RA5	ITS	*Trichoderma koningiopsis* strain 18ASMA001	MT520621.1	100	100
	*Tef1*	*Trichoderma koningiopsis* strain VSL155	MT058870.1	100	99.33
	*Rpb2*	*Trichoderma koningiopsis* isolate Tkois1	MT081443.1	100	99.60
UKM-M-UW RA6	ITS	*Trichoderma koningiopsis* strain 18ASMA001	MT520621.1	100	100
	*Tef1*	*Trichoderma koningiopsis* strain LESF360	KT278986.1	100	100
	*Rpb2*	*Trichoderma koningiopsis* isolate Tkois1	MT081443.1	100	100

**Table 4:** Details of genome assembly statistics of the three *Trichoderma* isolates RA3a, RA5, and RA6.

Parameter	RA3a	RA5	RA6
Number of contigs	14	11	13
Total contigs length	36,531,570	36,477,170	36,470,223
Mean contig size	2,609,397.86	3,316,106.36	2,805,401.77
Contig size first quartile	1,043,387	3,650,583	981,951
Median contig size	2,049,512	3,895,316	3,855,011
Contig size third quartile	5,555,030	6,876,866	5,268,312
Longest contig	6,903,293	6,995,056	6,877,006
Shortest contig	6075	6406	5219
Contigs > 500 nt	14 (100%)	11 (100%)	13 (100%)
Contigs > 1K nt	14 (100%)	11 (100%)	13 (100%)
Contigs > 10K nt	13 (92.86%)	10 (90.91%)	12 (92.31%)
Contigs > 100K nt	11 (78.57%)	8 (72.73%)	10 (76.92%)
Contigs > 1M nt	10 (71.43%)	7 (63.64%)	8 (61.54)
N50	5,555,030	5,554,967	3,979,290
L50	3	3	4
**N80**	2,447,863	3,862,469	3,855,011
**L80**	6	6	6

**Table 5:** Scaffold BUSCO: dataset Hypocreales odb10 for the genomes of the fungal isolates RA3a, RA5, and RA6.

BUSCO Scaffold Stat	RA3a	RA5	RA6
Percentage BUSCO	97.7%	97.7%	97.8%
Complete BUSCO’s	4392	4391	4394
Complete and single copy BUSCO’s	4378	4379	4381
Complete and duplicate BUSCO’s	14	12	13
Fragmented BUSCO’s	20	20	20
Missing BUSCO’s	82	83	80
Total BUSCO groups searched	4494		

**Table 6:** Proteins BUSCO: dataset Hypocreales_odb10 for the predicted proteomes of the fungal isolates RA3a, RA5, and RA6.

BUSCO Scaffold Stat	RA3a	RA5	RA6
Percentage BUSCO	92.3%	88.0%	87.4%
Complete BUSCO’s	4146	3955	3927
Complete and single copy BUSCO’s	4137	3946	3922
Complete and duplicate BUSCO’s	9	9	5
Fragmented BUSCO’s	108	219	245
Missing BUSCO’s	240	320	322
Total BUSCO groups searched	4494		

**Table 7:** Biosynthetic gene clusters predicted through AntiSMASH analysis for the genomes of the *Trichoderma* isolates RA3a, RA5, and RA6.

Fungal Strain	Total Clusters	NRPS-Like	PKS	Terpene	Hybrid NRPS/PKS	Hybrid PKS/Terpene
UKM-M-UW RA3a	43	17	14	7	4	1
UKM-M-UW RA5	40	16	11	8	4	1
UKM-M-UW RA6	41	17	12	7	4	1

## Data Availability

The genome sequences generated in this study were submitted to GenBank. BioSample metadata is publicly available in the NCBI BioSample database (http://www.ncbi.nlm.nih.gov/biosample/, last accessed on 25 January 2022) under accession numbers SAMN23731967, SAMN23731968, and SAMN23731969 for strains RA3a, RA5, and RA6 respectively. The annotated genomes are publicly available in the NCBI Genbank database (https://www.ncbi.nlm.nih.gov/genbank/, last accessed on 25 January 2022) under accession numbers JAJPEM000000000, JAJPEL000000000, and JAJPEK000000000 for strains RA3a, RA5, and RA6 respectively. The results of the StructRNAfinder analysis are publicly available at https://osf.io/vsbc2/ (last accessed on 25 January 2022).

## Supplementary Materials

The following are available online at https://www.mdpi.com/article/10.3390/jof8030246/s1, Table S1: Concentration of the extracted gDNA measured through Qubit. Figure S1: HMW genomic DNA of the three Trichoderma isolates. Table S2: Primers and PCR amplicon sizes for molecular identification of isolates. Table S3: Details on the number of predicted non-coding RNAs identified in each genome. Figure S2: Phylogenomic tree from alignment of 3392 single-copy orthologous proteins from selected Trichoderma spp. Figure S3: Shared and unique orthologous protein group among isolates sequenced in this study as inferred from OrthoVenn2 web server analysis. Table S4: Distribution of predicted BGCs found across the fungal genomes. Table S5: Putative salicylaldehyde cluster found across the three sequenced fungal genomes. Figure S4: Protein sequence comparison of the putative salicylaldehyde cluster found in the genome of strains RA6, RA5, and RA3a. Dataset S1: Genome analysis through eggnog mapper. Dataset S2: AntiSMASH analysis of genomes. Dataset S3: Details of secondary metabolite clusters conserved across the genomes of strains RA3a, RA5, and RA6.
