# Supplementary material for: Genome Sequencing and Analysis of Trichoderma (Hypocreaceae) Isolates Exhibiting Antagonistic Activity against the Papaya Dieback Pathogen, Erwinia mallotivora
Source: J Fungi (Basel). 2022 Feb 28;8(3):246. doi: 10.3390/jof8030246 (PMC8949440; doi:10.3390/jof8030246)
Supplement: Supplementary file 1 [file jof-08-00246-s001.zip › jof-1591442-SM/8. jof-1591442-final suppl/8.jof-1591442 - final-Supplementary Information.pdf]

Genome Sequencing and Analysis of *Trichoderma* (Hypocreaceae) Isolates Exhibiting Antagonistic Activity against the Papaya Dieback Pathogen, *Erwinia mallotivora*

Amin-Asyraf Tamizi[1], Noriha Mat-Amin[1], Jack A. Weaver[2], Richard T. Olumakaiye[2], Muhamad Afiq Akbar[3], Sophie Jin[2], Hamidun Bunawan[3,*] and Fabrizio Alberti[2,*]

[1] Agri-Omics and Bioinformatics Programme, Biotechnology and Nanotechnology Research Centre, Malaysian Agricultural Research and Development Institute Headquarters (MARDI), 43400 Serdang, Selangor, Malaysia;
[2] School of Life Sciences, University of Warwick, Gibbet Hill Road, Coventry CV4 7AL;
[3] Institute of Systems Biology, Universiti Kebangsaan Malaysia (UKM), 43600 Bangi, Selangor, Malaysia;
* Correspondence: hamidun.bunawan@ukm.edu.my; f.alberti@warwick.ac.uk

Supplementary Information

Table S1. Concentration of the extracted gDNA measured through Qubit.

Fungal isolate	DNA Concentration (ng/µl)
RA3a	127
RA5	168
RA6	103

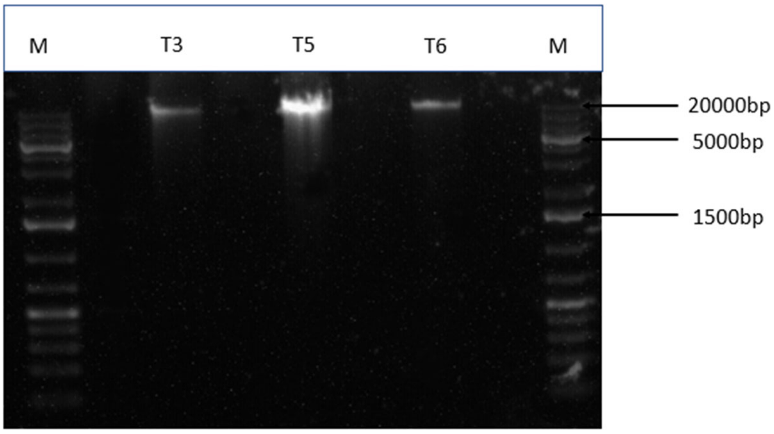

Figure S1. HMW genomic DNA of the three *Trichoderma* isolates. Lane M: GeneRuler 1 Kb Plus DNA ladder (Thermo Scientific). Lane T3: 1 µL of genomic DNA purified from isolate RA3a. Lane T5: 1 µL of genomic DNA purified from isolate RA5. Lane T6: 1 µL of genomic DNA purified from isolate RA6.

Table S2. Primers and PCR amplicon sizes for molecular identification of isolates.

**Marker**	**Forward primer**	**Primer sequence 5'->3'**	**Reverse primer**	**Primer sequence 5'->3'**	**Amplicon size (bp)**	**Reference**
Internal Transcribed Spacer (ITS)	ITS1F	CTTGGTCATTTAGAGGAAGTAA	ITS4	TCCTCCGCTTATTGATATGC	650	(Schoch et al., 2012)
Translation elongation factor 1 alpha (*tef1*) gene	EF1-728F	CATCGAGAAGTTCGAGAAGG	TEF1LLErev	AACTTGCAGGCAATGTGG	1,300	(Druzhinina and Kubicek, 2005)
RNA polymerase B subunit II (*rpb2*) gene	RPB2-5f	GAYGAYMGWGATCAYTTYGG	RPB2-7cR	CCCATRGCTTGYTTRCCCAT	1,200	(Liu et al., 1999)

Table S3. Details on the number of predicted non-coding RNAs identified in each genome.

Non-coding RNA's	RA3a	RA5	RA6
**Riboswitches**	1 (0.30%)	1 (0.29%)	1 (0.29%)
**Ribozymes**	2 (0.60%)	2 (0.59%)	2 (0.57%)
**Ribosomal RNAs (rRNA)**	89 (26.49%)	97 (28.53%)	101 (29.02%)
**Small nuclear RNAs (snRNA)**	9 (2.68%)	9 (2.65%)	9 (2.59%)
**Small nucleolar RNAs (snoRNA)**	31 (9.23%)	28 (8.24%)	30 (8.62%)
**Small RNAs (sRNA)**	4 (1.19%)	4 (1.18%)	4 (1.15%)
**Transfer RNAs (tRNA)**	195 (58.04%)	193 (56.76%)	196 (56.32%)
**Other**	5 (1.49%)	5 (1.47%)	5 (1.44%)
**intron**	0	1 (0.29%)	0

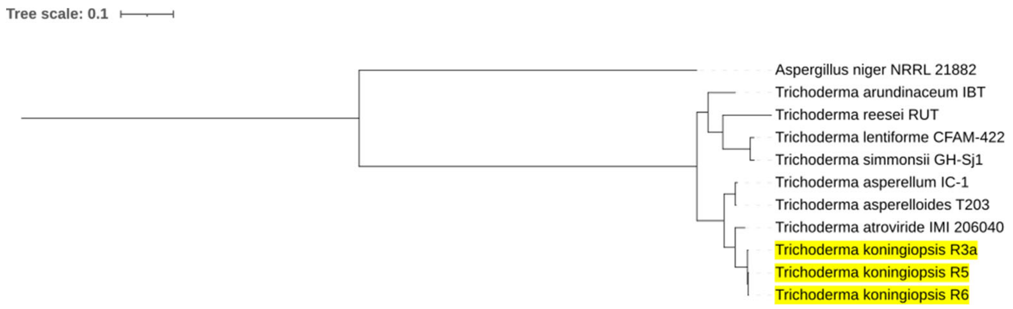

Figure S2. Phylogenomic tree from alignment of 3,392 single-copy orthologous proteins from selected *Trichoderma* spp. The three isolates sequenced in the present work are highlighted in yellow. Bootstrap values were omitted from the tree as all the branches showed 100% bootstrap value.

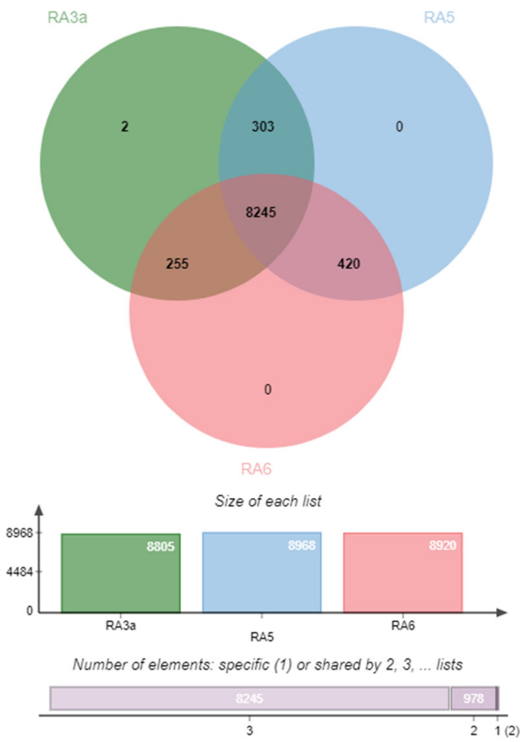

Figure S3. Shared and unique orthologous protein group among isolates sequenced in this study as inferred from OrthoVenn2 web server analysis.

Table S4. Distribution of predicted BGCs found across the fungal genomes.

**Gene Type**	**Fungal Genome**	**Region**	**Span**		**Cluster size (kb)**	**Most similar BGC according to AntiSMASH (% indicates the proportion of genes with similarities)**
			From	To		
**Hybrid PKS/NRPS**	RA3a	2.1	83,384	178,034	94.6	fusaric acid, 41% (5/12)
	RA5	7.3	3,470,945	3,555,885	84.9	fusaric acid, 50% (6/12)
	RA6	4.4	3,796,740	3,881,680	84.9	fusaric acid, 50% (6/12)
**PKS**	RA3a	6.1	78,041	120,092	42	naphthopyrone, 100% (1/1)
	RA5	5.9	3,799,152	3,841,850	42.6	naphthopyrone, 100% (1/1)
	RA6	5..9	3,806,331	3,849,029	42.6	naphthopyrone, 100% (1/1)
**PKS**	RA3a	6.3	725,578	749,075	23.4	neurosporin A, 100% (1/1)
	RA5	5.8	3,170,168	3,193,667	23.5	neurosporin A, 100% (1/1)
	RA6	5.8	3,180,784	3,200,851	20.0	neurosporin A, 100% (1/1)
**Hybrid PKS/Terpene**	RA3a	9.1	464,928	537,653	72.7	ascochlorin, 38% (3/8)
	RA5	1.6	6,455,881	6,532,090	76.2	ascochlorin, 38% (3/8)
	RA6	8.4	1,744,822	1,821,031	76.2	ascochlorin, 38% (3/8)
**Terpene**	RA3a	9.2	636,393	658,750	22.3	clavaric acid, 100% (1/1)
	RA5	1.4	6,335,790	6,356,639	20.8	clavaric acid, 100% (1/1)
	RA6	8.3	1,623,721	1,646,078	22.3	clavaric acid. 100% (1/1)

Table S5. Putative salicylaldehyde cluster found across the three sequenced fungal genomes.

**Gene Locus**	**Homologue (% Identity/% similarity), Organism, Accession number**	**Proposed function**
**RA3a_007616, RA5_007274, RA6_006441**	Highly reducing polyketide synthase *virA*, 84%/92%, *Trichoderma virens* Gv29-8, G9N4B2.1	Biosynthesis of the polyketide unit
**RA3a_007617, RA5_007273, RA6_006440**	Short-chain dehydrogenase virB, 75%/81%, *Trichoderma virens* Gv29-8, G9N4B1.1	Tailoring of the scaffold
**RA3a_007618, RA5_007272 RA6_006439**	Cupin-domain-containing oxidoreductase *virC,* 88%/95%, *Trichoderma virens* Gv29-8, G9N4B0.1	
**RA5_007271**	Short-chain dehydrogenase virD, 62%/67%, *Trichoderma virens* Gv29-8, G9N4A9.1	
**RA3a_007619, RA5_007270, RA6_006438**	Cytochrome P450 monooxygenase virE, 74%/83%, *Trichoderma virens* Gv29-8, G9N4A8.1	
**RA3a_007620, RA5_007269, RA6_006437**	Adenylosuccinate lyase, 49%/63%, *Saccharomyces cerevisiae* S288C, Q05911.1	
**RA3a_007621, RA5_007268, RA6_006436**	Probable oxidoreductase virH, 81%/89%, *Trichoderma virens* Gv29-8] Sequence ID: G9N4A5.1	
**RA3a_007622, RA5_007267, RA6_006435**	Glutamine--tRNA ligase protein *virJ*, 90%/93%, *Trichoderma virens* Gv29-8 G9N4A3.1	
**RA3a_007623, RA5_007266**	FAD-linked oxidoreductase virF, 59%/73%, *Trichoderma virens* Gv29-8, G9N4A7.1	

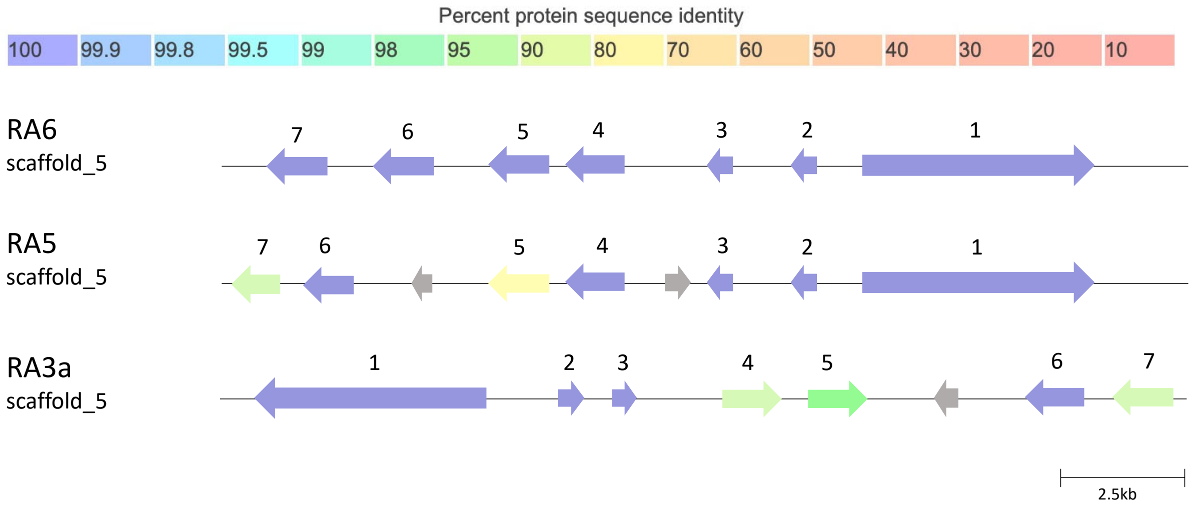

Figure S4. Protein sequence comparison of the putative salicylaldehyde cluster found in the genome of strains RA6, RA5, and RA3a. The proteome comparison service from PATRIC

3.6.12 (Davis et al., 2020) was used to carry out protein sequence comparison using bi-directional BLASTP. Default parameters were used; i.e Minimum % coverage was set to 30%, BLAST E value was set to 1e-5 and Minimum % Identity was set to 10%. The protein sequence of the RA6 cluster was used as a reference sequence to be compared to the RA5 cluster and the RA3a cluster. The sequences shown in grey are sequences that did not show any similarity between clusters. Key: 1, HR-PKS; 2, Short chain dehydrogenase; 3: Cupin-domain-containing oxidoreductase; 4, Cytochrome P450; 5, Lyase; 6, Oxidoreductase; 7, Oxidoreductase.

References

Davis, J.J., Wattam, A.R., Aziz, R.K., Brettin, T., Butler, R., Butler, R.M., Chlenski, P., Conrad, N., Dickerman, A., Dietrich, E.M., Gabbard, J.L., Gerdes, S., Guard, A., Kenyon, R.W., Machi, D., Mao, C., Murphy-Olson, D., Nguyen, M., Nordberg, E.K., Olsen, G.J., Olson, R.D., Overbeek, J.C., Overbeek, R., Parrello, B., Pusch, G.D., Shukla, M., Thomas, C., VanOeffelen, M., Vonstein, V., Warren, A.S., Xia, F., Xie, D., Yoo, H., Stevens, R., 2020. The PATRIC Bioinformatics Resource Center: expanding data and analysis capabilities. Nucleic Acids Res. 8, D606-D612. doi: 10.1093/nar/gkz943.

Druzhinina, I., Kubicek, C.P., 2005. Species concepts and biodiversity in Trichoderma and Hypocrea: From aggregate species to species clusters. J. Zhejiang Univ. Sci. 6 B, 100–112. https://doi.org/10.1631/jzus.2005.B0100

Liu, Y.J., Whelen, S., Hall, B.D., 1999. Phylogenetic relationships among ascomycetes: Evidence from an RNA polymerse II subunit. Mol. Biol. Evol. 16, 1799–1808. https://doi.org/10.1093/oxfordjournals.molbev.a026092

Schoch, C.L., Seifert, K.A., Huhndorf, S., Robert, V., Spouge, J.L., Levesque, C.A., Chen, W., Bolchacova, E., Voigt, K., Crous, P.W., Miller, A.N., Wingfield, M.J., Aime, M.C., An, K.D., Bai, F.Y., Barreto, R.W., Begerow, D., Bergeron, M.J., Blackwell, M., Boekhout, T., Bogale, M., Boonyuen, N., Burgaz, A.R., Buyck, B., Cai, L., Cai, Q., Cardinali, G., Chaverri, P., Coppins, B.J., Crespo, A., Cubas, P., Cummings, C., Damm, U., de Beer, Z.W., de Hoog, G.S., Del-Prado, R., Dentinger, B., Diéguez-Uribeondo, J., Divakar, P.K., Douglas, B., Dueñas, M., Duong, T.A., Eberhardt, U., Edwards, J.E., Elshahed, M.S., Fliegerova, K., Furtado, M., García, M.A., Ge, Z.W., Griffith, G.W., Griffiths, K., Groenewald, J.Z., Groenewald, M., Grube, M., Gryzenhout, M., Guo, L.D., Hagen, F., Hambleton, S., Hamelin, R.C., Hansen, K., Harrold, P., Heller, G., Herrera, C., Hirayama, K., Hirooka, Y., Ho, H.M., Hoffmann, K., Hofstetter, V., Högnabba, F., Hollingsworth, P.M., Hong, S.B., Hosaka, K., Houbraken, J., Hughes, K., Huhtinen, S., Hyde, K.D., James, T., Johnson, E.M., Johnson, J.E., Johnston, P.R., Jones, E.B.G., Kelly, L.J., Kirk, P.M., Knapp, D.G., Kõljalg, U., Kovács, G.M., Kurtzman, C.P., Landvik, S., Leavitt, S.D., Liggenstoffer, A.S., Liimatainen, K., Lombard, L., Luangsa-ard, J.J., Lumbsch, H.T., Maganti, H., Maharachchikumbura, S.S.N., Martin, M.P., May, T.W., McTaggart, A.R., Methven, A.S., Meyer, W., Moncalvo, J.M., Mongkolsamrit, S., Nagy, L.G., Nilsson, R.H., Niskanen, T., Nyilasi, I., Okada, G., Okane, I., Olariaga, I., Otte, J., Papp, T., Park, D., Petkovits, T., Pino-Bodas, R., Quaedvlieg, W., Raja, H.A., Redecker, D., Rintoul, T.L., Ruibal, C., Sarmiento-Ramírez, J.M., Schmitt, I., Schüßler, A., Shearer, C., Sotome, K., Stefani, F.O.P., Stenroos, S., Stielow, B., Stockinger, H., Suetrong, S., Suh, S.O., Sung, G.H., Suzuki, M., Tanaka, K., Tedersoo, L., Telleria, M.T., Tretter, E., Untereiner, W.A., Urbina,

H., Vágvölgyi, C., Vialle, A., Vu, T.D., Walther, G., Wang, Q.M., Wang, Y., Weir, B.S., Weiß, M., White, M.M., Xu, J., Yahr, R., Yang, Z.L., Yurkov, A., Zamora, J.C., Zhang, N., Zhuang, W.Y., Schindel, D., 2012. Nuclear ribosomal internal transcribed spacer (ITS) region as a universal DNA barcode marker for Fungi. Proc. Natl. Acad. Sci. U. S. A. 109, 6241–6246. https://doi.org/10.1073/pnas.1117018109
